# Memory Integration as a Challenge to the Consolidation/Reconsolidation Hypothesis: Similarities, Differences and Perspectives

**DOI:** 10.3389/fnsys.2018.00071

**Published:** 2019-01-11

**Authors:** Pascale Gisquet-Verrier, David C. Riccio

**Affiliations:** ^1^Institut des Neurosciences Paris-Saclay (Neuro-PSI), Université Paris-Sud, CNRS UMR 9197, Université Paris-Saclay, Orsay, France; ^2^Department of Psychological Sciences, Kent State University, Kent, OH, United States

**Keywords:** memory, consolidation, reconsolidation, reactivation, retrograde amnesia, state-dependent memory, updating, false memory

## Abstract

We recently proposed that retrograde amnesia does not result from a disruption of the consolidation/reconsolidation processes but rather to the integration of the internal state induced by the amnesic treatment within the initial memory. Accordingly, the performance disruption induced by an amnesic agent does not result from a disruption of the memory fixation process, but from a difference in the internal state present during the learning phase (or reactivation) and at the later retention test: a case of state-dependency. In the present article, we will review similarities and differences these two competing views may have on memory processing. We will also consider the consequences the integration concept may have on the way memory is built, maintained and retrieved, as well as future research perspectives that such a new view may generate.

## The Consolidation Hypothesis

### Original Findings and Interpretation

There have been hundreds of publications describing the findings that treatments delivered after training or after the reactivation of a memory disrupt the retention performance. The disruption is time dependent: greater when the treatments are delivered shortly after training or reactivation rather than long after. These findings have been reported in many species, and after various tasks. Treatments used to disrupt the retention performance have changed over time. Initially disruptive treatments were severe treatments delivered systemically because they were believed to alter brain functioning. These included manipulations such as electroconvulsive shock, hypothermia, hypercapnia, anesthesia, spreading cortical depression…. Simultaneously, and at approximately the same period, other treatments were shown to increase the retention performance with similar time dependent effects. In that case, treatments such as strychnine, amphetamine were presumably stimulating brain functioning. All these results have been interpreted as resulting from effects on a time-dependent process required to progressively fix the memory in a stabilized form that has been termed consolidation (Glickman, 1961; McGaugh, 1966). This view supported previous suggestions such as those proposed by Hebb (1949) who proposed that reverberating circuits may take place when a new information is presented. Such a notion was close to the idea previously developed by Müller and Pilzecker (1900) whose work on retroactive interference led them to suggest that new memories are fragile, requiring time to undergo a consolidation phase before being fixed.

Disrupting this process by treatments interfering with the normal brain functioning was thus supposed to prevent memory formation and to lead to amnesia. Mirroring this logic, facilitative treatments were supposed to strengthen this process. The length of the consolidation period was thus defined by the time after which the administration of the treatment no longer affected the retention performance. Depending on numerous parameters such as the task, the strength of initial training, and intensity of the amnesic treatment, the duration largely varied, but was considered to be roughly between 30 min and 2 h.

According to that scheme, memory should be permanently established and should no longer be susceptible to an amnesic treatment. However both of these assertions have been demonstrated to be wrong. First, several studies reported that amnesia may spontaneously recover (Cooper and Koppenaal, 1964; Squire and Barondes, 1972). Some other studies indicated that memory impairments induced by some amnesic treatments can be reversed by other drugs administered near the time of training (Martinez et al., 1981). Finally, there is extensive evidence, much of it before 1980, showing that delivering reminder cues such as the unconditioned stimulus used during initial training or even the motivational state, shortly before the retention test, were able to abolish retrograde amnesia (Misanin et al., 1968; Robbins and Meyer, 1970; Miller and Springer, 1972; Mactutus et al., 1979). More intriguingly, some studies even reported that the re-administration of the “amnesic” treatment before the retention test could also alleviate or reverse the memory loss. This has been demonstrated with several of them, including ECS, hypothermia, protein synthesis inhibition and cortical spreading depression (Thompson and Neely, 1970; Greenwood and Singer, 1974; Hinderliter et al., 1975; Bradley and Galal, 1988). Up to now, these results have never been satisfactorily explained by the consolidation hypothesis.

Second, during the same period, research showed that retrograde amnesia could be obtained on an already fixed memory, provided that animals were previously exposed to a “reminder” that served to reactivate the stored information (Miller and Springer, 1972; Gordon and Mowrer, 1980). Such a result gave rise to an important notion introduced by Lewis (1969, 1979), namely that the memory may exist in two different forms. One would be an active form characterizing newly acquired information or old but reactivated, memory, susceptible to external alterations. The other would be an inactive form, typical of stored but potentially accessible memories, that would not be affected by any amnesic treatments.

Another finding, also published around the same time, indicated that the disruption of performance was not detectable during the first few hours, but occurred only later (Geller et al., 1970; Miller and Springer, 1971, 1972; Hinderliter et al., 1975). Such a delayed onset of amnesia forced people supporting the consolidation hypothesis to suggest new trainings were stored both in a short and in a long-term store, and that consolidation process only concerns with only the long-term one (McGaugh, 1966; Miller and Matzel, 2000). The same should be proposed after memory reactivation.

### An Alternative Interpretation

In fact there is another way to explain the results supporting the consolidation hypothesis. We recently proposed that the performance disruption resulting from various treatments delivered after a new learning does not result from a storage alteration but from the integration within the memory of the internal state induced by the treatment (Gisquet-Verrier and Riccio, 2018). Since “amnesic” treatments are always severe, the resulting alteration of the internal state constitutes a very salient cue. The absence of this cue at the time of testing would be responsible for retrieval difficulties. However, helping the retrieval processes, through the exposure to reminders (with reintroducing the state induced by the amnesic treatment being a very effective cue) could compensate for the impairment. Such an explanation in terms of state dependency, which had been proposed as soon as in 1975 (Hinderliter et al., 1975), can account for all the results previously reported, including those that do not fit with the consolidation view, previously noted. State dependent retention has long been recognized but has been experimentally investigated extensively following a study reported in 1964 by Overton (1964). The term refers to a performance disruption resulting from a change in experimental conditions between training and testing. State-dependency has been demonstrated with different types of changes including either the external or the internal context, but mainly with various kinds of drugs, including alcohol, morphine, amphetamine…. always delivered before training (see Radulovic et al., 2017, for a review). The main difference between the traditional state dependency view and retrograde amnesia is that in this latter situation, the treatment is delivered after training and not before.

It is noteworthy that when “amnesic” treatments are delivered before training, a time-dependent disruption of performance, termed anterograde amnesia, is also observed (Gruber et al., 1967; Kesner, 1971) and state dependency has been considered as responsible for anterograde amnesia (e.g., Richardson et al., 1984).

Hence, there is evidence indicating that the state induced by treatments delivered just before or after a training episode is integrated within the memory of that episode and becomes part of the retrieval cues for that episode. We thus propose that the newly formed memory is malleable and susceptible to the integration of new information during a time window overlapping the time of training itself (see Gisquet-Verrier and Riccio, 2018).

### Current Views on the Consolidation Hypothesis

We have briefly presented evidence indicating that state-dependency may well explain the first results supporting the consolidation hypothesis as well as those which were not predicted by the hypothesis. Of course, during the period following 1975–1980, the consolidation hypothesis largely evolved but remained based on the same findings, mainly retrograde amnesia. The consolidation hypothesis has been compared with the phenomenon of long-term potentiation (LTP), a model of synaptic plasticity supposed to play a determinant role in memory formation. Similarities between consequences of learning and LTP, which drove research interests toward cellular and molecular explanations of learning and memory, appeared to strengthen the consolidation hypothesis and overshadowed the serious criticisms concerning the consolidation hypothesis (Bruel-Jungerman et al., 2007; Takeuchi et al., 2013; but see Shors and Matzel, 1997). These studies led to the contention that new connections within the neuronal network involved during training constituted the support for the memory, suggesting that the synthesis of new protein synthesis was essential for the formation of new memories. As a consequence, treatments used to induce retrograde amnesia were essentially protein synthesis inhibitors or substances interfering with the molecular cascades leading to protein synthesis (Nader and Hardt, 2009; Hardt et al., 2010; Lee et al., 2017). However, it has been shown that re-administering a protein synthesis inhibitor before the retention test is able to abolish retrograde amnesia (Briggs and Olson, 2013; Gisquet-Verrier et al., 2015). The way treatments were delivered also changed, as they are now frequently directly injected within brain structures such as the amygdala or the hippocampus, which have been shown to be important for the establishment of memory. However, state dependency is a very sensitive phenomenon and can be obtained with drugs delivered not only systemically but also with drugs delivered in a small amount, within specific brain regions (Jamali-Raeufy et al., 2011; Rossato et al., 2015; Radulovic et al., 2017).

In 2000, a famous study re-introduced the finding discovered 30 years before, namely that remote memory can be made susceptible again to an amnesic agent provided that the initial memory was previously reactivated. However, this experiment was performed in a more sophisticated analytic way than earlier studies and gave rise to the reconsolidation hypothesis (Nader et al., 2000; see also Przybyslawski and Sara, 1997; Sara, 2000). Memory reconsolidation presumably consists of two phases, a reactivation-dependent destabilization process, followed by the protein synthesis-dependent restabilization phase. According to this hypothesis, retrieved memories can re-enter into a state of lability, requiring processes close to those involved during the consolidation, in order to re-stabilize the memory. Such an assertion is based on studies showing that reactivated memories are susceptible again to the same treatments as newly acquired memories. Is this phenomenon possibly due to a state-dependency? Two different studies support this position. First, it has been shown that the retention of a contextual fear can be affected by the internal state in which the reactivation occurs. When the reactivation cue is presented while the animals are water deprived, the retention performance is disrupted unless the same sate is re-introduced at the time of testing (Sierra et al., 2013). Also, in a recent study we showed that the administration of cycloheximide, a protein synthesis inhibitor, or of lithium chloride, a substance inducing strong nausea, delivered just after the reactivation of an inhibitory avoidance memory, induced a strong performance disruption, which was abolished by delivering again the same treatment before the retention test (Gisquet-Verrier et al., 2015). These studies indicated that the malleability period, which characterized newly formed memories, can be reinstated during memory reactivation. These findings strongly suggest that after reactivation, just as after training, new information can be inserted into a memory.

## State Dependency to Explain Retrograde Amnesia

### How State Dependency Can Explain Delayed Onset of Retrograde Amnesia

According to the state dependency hypothesis, retention performance should not be affected when the subject is in the same internal state than the one prevailing at the time of training or shortly thereafter. As a consequence, the disruption of performance would only appear when the internal state returns to a basal level, i.e., several hours after the administration of the treatment. Such a view nicely accounts for the delayed onset of amnesia, which has been evidenced after training and after memory reactivation.

### How State Dependency Can Explain Recovery of Amnesia

As previously mentioned, state-dependency disrupts retrieval because some of the main cues triggering retrieval processes are not present at the time of testing. Such a view explains why providing help for retrieval in the form of exposure to reminders may abolish the performance disruption. It is noteworthy that the most efficient cue is to re-introduce the drug state (or state induced by amnesic treatments) before testing.

### Relationship of Certain Training Conditions to State Dependency

Several other findings that seem inconsistent with the consolidation view may in fact be compatible with that state dependent interpretation. For instance, it has been noted that several factors, including a strong training reinforcer, overtraining, pre-exposure to the conditioning context, or weak amnestic treatments, can reduce the susceptibility to retrograde amnesia (Geller et al., 1970; Miller, 1970; Mactutus et al., 1982; Parent and McGaugh, 1994; Garín-Aguilar et al., 2014). We suggest that when the information acquired during initial training is unusually strong, due to the use of numerous training trials or of a strong reinforcer, the internal state produced by the amnestic agent is less salient than the training state. Thus, its absence at testing would not affect retrieval. Similarly, if the treatment itself induces only a slight alteration of the internal state, the absence of that context at the time of testing would be irrelevant to the retrieval process.

## The Concept of Memory Integration

We have provided evidence indicating that state dependency can explain retrograde amnesia. However, state dependency cannot be considered as an alternative to the consolidation hypothesis, because as previously noted by several authors, state dependency cannot account for all the results supporting the consolidation view. These results include the effects of treatments enhancing the retention performance and those interfering with the initial training, which have been frequently used to support the existence of reconsolidation in humans (Nader and Hardt, 2009; Lee et al., 2017).

However, we must emphasize that state dependency in experiments on retrograde amnesia is the result of the integration of a modified state within the initial memory, while in an active state, that is malleable^1^. Thus, it is the integration concept, which actually challenges the consolidation hypothesis.

### Memory Integration: The Concept

Memories are dynamic and must be able to be modified over time in order to be updated. This process must be easy and rapid. Every time we need to update a memory, a reactivation of that memory takes place, putting it in an active state (Lewis, 1979; Gisquet-Verrier and Riccio, 2012). The main characteristic of an active memory is its malleability, i.e., its ability to integrate new information. The malleability window extends over a period running from shortly before to shortly after reactivation. Any information presented during that time window can be integrated with that memory. The new information does not replace the previous information because it is important to keep track of our memories along a time scale. However, it may happen that the new information prevails over an older one. While it is the case that many memory investigators would agree with the concepts of malleability and integration, our issue here is with respect to the venerable consolidation hypothesis.

The role of memory integration is mainly to update memories, a process required to actualize our knowledge about the world during the lifespan. However, the basic and fundamental process can be hijacked in some circumstances, as in the case of retrograde amnesia (Gisquet-Verrier and Riccio, 2018).

### Case of Facilitative Treatments

The fact that some treatments administered soon after an experience enhance the retention performance and do so in a retrograde time-dependent manner, has been taken as evidence supporting the consolidation hypothesis (McGaugh, 1966; Hardt et al., 2009; Lee et al., 2017). It is generally considered that these treatments have the opposite effects of those attributed to the amnesic treatment: to strengthen the consolidation process. However, the way by which these treatments may operate still remains an open question. Broadly speaking, treatments leading to an improvement of the retention performance are less numerous than those disrupting the retention performance. The more usual are treatments known to stimulate the nervous system such as glucose, amphetamine, strychnine, and reticular activation (Gold and Van Buskirk, 1975; McGaugh, 1966). Accordingly, one may consider that all these treatments stimulate brain activity, while the memory is in an active state. Interestingly, some of these treatments have been successfully administered after amnesic treatments to decrease the memory impairment (Martinez et al., 1981). We propose that treatments known to improve the subsequent retention are likely to do so by enhancing arousal, a technique well known to produce such an outcome (Bloch et al., 1970). Since these effects are more pronounced when the increase of arousal occurs closely in time with training, such a view may account for the time dependent effects of the promnesic treatments. Some studies support this assertion; for instance, it has been reported that an electrical stimulation of the reticular formation, known to induce arousal, was able to facilitate the retention performance in a time-dependent fashion, when delivered both after training and after memory reactivation (Deweer et al., 1968; DeVietti and Kirkpatrick, 1977). Accordingly, unlike the consolidation hypothesis which analyses retrograde amnesia and facilitative effects as resulting from two opposite causes, namely disrupting or enhancing the consolidation process, we propose that both effects result from the same process: the integration of a new information within the memory which leads to two opposite outcomes.

### Memory Interference

Since 2000, the question of whether reconsolidation could be evidenced in human has become an important issue. Since, due to their toxicity, most of the agents blocking reconsolidation cannot be used in humans, the idea of disrupting the reconsolidation processes in a more acceptable way emerged. This has been achieved by the use of retroactive interference. Learning a task shortly after the acquisition of a similar one generally induces performance disruption in the retention of the former one. Since interaction between the initial memory while in an active state and the new one is analyzed as responsible for the disruption, retroactive interference has been taken has a possible way to disrupt reconsolidation process and hence to provide experimental support for the existence of this process in humans. First demonstrated for procedural memory (Walker et al., 2003), retroactive interference has also been demonstrated for episodic memory with the use of a paradigm based on lists of words (Hupbach et al., 2007). However, these studies have been recently brought into question (Hardwicke et al., 2016; Klingmüller et al., 2017), and it should be emphasized that in the paradigm introduced by Hupbach et al. (2007) the disruption of performance mainly results from the intrusion of items of the second list into the memory of the first list, and not from a forgetting of the first list of words (see Scully et al., 2017). This finding is thus more in agreement with the integration concept than with the consolidation hypothesis.

### Boundary Conditions

Despite the fact that the state dependency hypothesis has not often been systematically tested in studies reporting retrograde amnesia, we must acknowledge that some studies failed to find the state dependency effect (Thompson and Neely, 1970; DeVietti and Larson, 1971; Lee et al., 2004; Garín-Aguilar et al., 2014). However, negative results are difficult to interpret unless a wide range of parameters (e.g., dosage, time between 2nd administration and testing, etc.). Moreover, it should be noted that cases of state dependency have been reported for almost of types of amnesic agents, even occasionally by people failing to obtain it in other circumstances.

The integration concept proposes to account for the promnesic effect of drugs by their arousal properties. Although this seems to be the case for many of them, we acknowledge that others such as acetylcholine, serotonin, estradiol and melanocortin peptides (de Wied, 1997; ter Horst et al., 2012), are not classically considered as arousal enhancers. More investigations are thus required to extend our hypothesis to all facilitative treatments.

State dependency is a hypothesis which can be considered for any treatment delivered before or after training. Taking this possibility into account may introduce important changes in our understanding of memory processes. In cases where the memory was never acquired (stored) initially, as with pre-training lesions, the concept of state dependency would not be relevant, but it can be evoked for post training lesions as well as for transitory blockade delivered either shortly before or shortly after training.

There are a few findings in the literature showing that more than one period is sensitive to disruption of memory formation after acquisition (Bernabeu et al., 1997; Bourtchouladze et al., 1998; Trifilieff et al., 2006). Other studies mention that memory disruption can be obtained by treatments delivered long after training (several hours), within specific brain regions such as insular cortex. We agree that these results cannot be explained in terms of state dependency, but they also cannot be explained by the traditional consolidation hypothesis.

## Integration Vs. Consolidation: What Changes?

A question often raised with respect to the integration concept is: but what is really new with the integration concept? Basically, our answer is that integration leads to a very different conception of memory and it is thus important to elaborate that particular topic.

### Malleability of Active Memory

**Figure 1:**
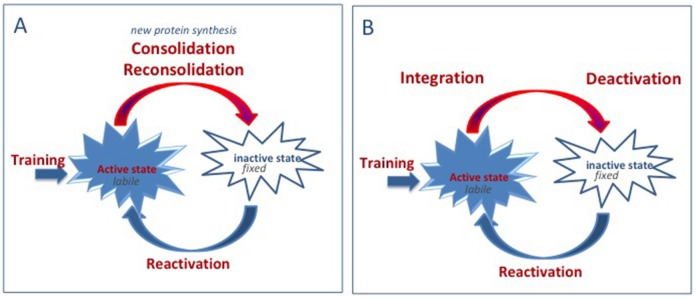
Comparison of the consolidation/reconsolidation hypothesis and the integration concept. **(A)** Consolidation/reconsolidation hypothesis: when reactivated, the memory is in an active state. When active, memories are labile/fragile and can be disrupted. Consolidation or reconsolidation process is required to fix/stabilize new and reactivated memories and to update remote memories. These processes take time and require new protein synthesis. Disruption of these processes leads to retrograde amnesia. **(B)** Integration concept: when reactivated, the memory is in an active state. When active, memories are labile and can integrate new information. Depending on the information content, integration could either result in updating (new/complementary information), strengthening (trials, “promnesic” treatments), disrupting (“amnesic” treatment, interference), or distorting (counterconditioning, false information) the initial memory.

The proponents of the two positions both consider that memory can be defined as existing in one of two different states. The active state, which characterizes the memory at the time of training and during the reactivation of the memory, and the inactive state during which the information is present but not directly accessible. They also agree on the assertion that, when active, the information content of a memory is available and malleable. However, for people supporting the consolidation hypothesis, malleability means fragility, that is, the failure to encode memory as the result of an amnesic treatment. On the other hand, malleability for us refers to flexibility, to the capacity to integrate new information. In addition, we have provided evidence indicating that the period of malleability is not restricted to the time just after conditioning/reactivation, but can be extended to a longer time period, which includes (backwards) times preceding training/reactivation (Miller et al., 1989). Following reactivation, the memory becomes less and less malleable. This progressive period of deactivation is responsible for the progressively decreasing effects of interfering treatments, including amnesic and promnesic treatments (see Figure 1). According to the integration concept, there is no stabilization or destabilization but there are circumstances during which a memory is malleable and others where it is not. There is no time-dependent process beginning immediately after learning during which a memory is susceptible to disruption, but there is a period of malleability during which all the information present in the environment can be combined to form a modified memory. Such a point of view has been illustrated some years ago by a series of experiments indicating the possibility of strengthening a memory by delivering a novel and unrelated experience during a time window extending from before to after training. This process had been termed behavioral tagging and is thought to be supported by synaptic tagging (Moncada and Viola, 2007; Redondo and Morris, 2011). More recently, the same idea has resurfaced in convincing studies demonstrating the possibility of linking two unrelated memories, provided that they were delivered closely in time to each other (Cai et al., 2016; Rashid et al., 2016). Hence, the malleability of active memory appears to be the very basis of the dynamic quality of memories, allowing the possibility to link, to complete, to modify and to update recent and remote memories.

### Processing of New Information

Following their initial acquisition, memories should be able to be modified through further experience. Consolidation and integration hypotheses both consider that updating requires the reactivation of the initial memory. However, for the tenets of the consolidation hypothesis, updating occurs through a time-dependent reconsolidation process (Lee, 2008; Hardt et al., 2010). They further hold that two different types of information must be considered. First, treatments delivered when a memory is malleable are presumed to interfere with the consolidation/reconsolidation process, preventing (or strengthening) the stabilization of the memory and leading to a performance disruption (or enhancement). Second, information such as exposure to contextual or sensory stimuli, electrical shocks, or new trials can potentially integrate the active memory for an updating.

According to the integration concept, the establishment of the memory itself is very rapid and all information, provided it is salient enough, presented when a memory is active can be integrated into that memory. However, depending on its content, the consequence of that integration may have different outcomes (see Figure 1). It may lead to a disruption of the retention performance, when the added information introduces new cues that will not be present at the time of testing (such as amnesic treatments), or cues that are sources of confusion through competing memories (interference). New information can also distort the initial memory when incorrect information is presented (false memory). However, new information can also improve the memory when consistent information is presented (retraining), or lead to the formation of a new memory (rule shifting, extinction, new association). The integration concept proposes that when malleable, all significant information surrounding an event will be part of that memory, with various consequences depending on the information content.

### Memory Is Not Erased but Can Be Modified

According to the consolidation/reconsolidation hypothesis, treatments delivered just after training/reactivation interfere with the stabilization/re-stabilization processes, preventing the storage/re-storage of information. As a consequence, new memories and reactivated memories are considered “lost” or prevented from being established (Dudai and Eisenberg, 2004; Hardt et al., 2010; Lee et al., 2017). The clear implication is that the information has, in some sense, been “erased.” This is in contrast with the integration concept, which considers that memories are not eliminated but can be modified with the help of new information proposed while the memory is in an active state. Most of the time, new information is used to update memory, an essential process in actualizing our current knowledge. However, the updating process can be diverted from its normal objective for other purposes and may lead to the formation of false memories. It seems relatively easy to change people’s memory of the details of an event that they have actually experienced. Research showed that creating false memories of a relatively benign childhood experience, such as becoming lost in a shopping mall as a young child, can be induced (Loftus, 1996, 2005). We propose that memories can easily be modified, enlarged with new components and that this basic updating process can be diverted to implant false memory.

### Do Memories Require New Protein Synthesis?

The consolidation hypothesis emphasizes the central role of new protein synthesis in the stabilization or the re-stabilization of memories, a view that has been extensively documented in the literature (Abel and Lattal, 2001; Tronson and Taylor, 2007; Besnard et al., 2012). However, the role of new proteins in the formation of memories has often been questioned in the past (Squire and Barondes, 1972; Gold, 2008). But since it has been considered that the neural network activated by an event constitutes the support of its representation in the brain, the implication of new protein synthesis in memory formation seems mandatory. Nevertheless, numerous findings indicate that behavioral, electrophysiological and pharmacological manipulations can rescue memory (and LTP) from the disruption induced by protein synthesis inhibition (see Gold, 2008, for more details). We recently showed that even when more than 80% of the protein synthesis is blocked, new memories can be formed and retrieved, provided that some help for retrieval is provided (Gisquet-Verrier et al., 2015). Interestingly in another study published the same year, Tonegawa’s lab, using optogenetic techniques targeting directly the neural network involved during conditioning came to the same conclusion (Ryan et al., 2015). However, they did not rule out the participation of new protein synthesis and proposed that, contrary to what is generally considered, new proteins might not be necessary for memory formation but rather for memory retrieval (Ryan and Tonegawa, 2016). Nevertheless, this position seems unlikely for two main reasons. First, because this hypothesis does not explain why delivering again a protein synthesis inhibitor before testing could help for retrieval. Second, this hypothesis seems also unable to explain why the inhibition of protein synthesis after reactivation may prevent successful retrieval since protein synthesis presumably occurred at the time of original training (Gisquet-Verrier and Riccio, 2016). So we propose that encoding and retrieving memories properly do not require new protein synthesis.

### Neuronal Networks May Not Constitute the Support of Memory Traces

There is substantial evidence indicating that training induces alterations within the neuronal network activated by the training situation. Activated neurons have been found to undergo chemical changes, through complex molecular cascades, increasing the synaptic strength and leading to the formation of new synaptic connections and reorganization of existing ones (Tronson et al., 2006). These synaptic and the cellular changes, which obviously depend on the training episode and occur during the consolidation time period, have thus been considered as demonstrating that the neuronal network engaged during training supported the memory. If we consider that new memories do not require new protein synthesis, as proposed by several authors (Gold, 2008; Gisquet-Verrier et al., 2015; Ryan et al., 2015), the hypothesis that the neuronal network engaged during training may constitute the support of the memory trace is seriously questioned. Here again the results supporting the initial proposition are not called into question, but their interpretation can be revised. This raises the question of knowing what is responsible for synaptic changes in an activated network. It is generally thought that the changes result from training, that is from the information content, but there is another possibility: the changes could be due to the activation of the network *per se*, irrespective of what circumstances activated the network. If this is correct, synaptic changes should be obtained after any event stimulating the brain, even those not related to a training episode. Although this proposal needs further investigation, it has been shown that changes similar to those seen after training can be obtained after an electroconvulsive shock (Stewart and Reid, 1994; Reid and Stewart, 1997), after an epileptic crisis (Meador, 2007) or after a specific or non-specific brain stimulation (Ma et al., 2013; Lee et al., 2014). In other words, it appears that synaptic plasticity may result from the activation of the network rather than by the sources of the activation. Recent findings obtained with techniques such as optogenetic manipulations seem to support the notion that the associated neural network supports the memory trace. It has been repeatedly shown that the stimulation of a particular part of the brain circuit, such as the hippocampal engram cells, can trigger the reactivation of a complex memory such as a contextual fear memory in which these neurons have been involved (Ryan et al., 2015; Tonegawa et al., 2018). This finding, however, cannot be taken as the demonstration that the tagged neurons are part of the memory representation. We have known for a long time that delivering a shock has the same effect as stimulating brain regions eliciting pain (Maho and Bloch, 1992) and that central stimulation concerning sensory or associative structures can substitute for a CS (Mouly et al., 1990; Doyère and Laroche, 1992). Since memory reactivation can be triggered by the exposure to a reminder cue, it is likely that a similar effect could be obtained by the stimulation of the brain circuit activated by these cues, or even by the stimulation of brain structures involved in the association between cues. Hence, we suggest that memory representations are not supported by the neural network activated by the training episode (see Queenan et al., 2017 for other alternatives).

### Memories Are Rapidly Formed and Can Be Rapidly Modified

According to the consolidation hypothesis, the fixation of a new memory requires time. The length of the consolidation process has been estimated by the duration of the susceptibility to amnesic treatments. This length appears to be highly variable but can range from minutes to hours depending on numerous factors such as the task, the treatment, the level of training, etc. (e.g., Dudai, 2012). However, some studies suggested that consolidation may extend over much more longer periods of time (see comments under boundary conditions above). It may seem curious that our sophisticated brain takes so much time to register information that may be critical for the survival of individuals. It is also surprising that memories are fragile for an extended period of time, not only during their initial formation, but also every time memories are retrieved. Finally, it is very surprising that such a scheme does not result in more frequent amnesia than those generally observed in human. The integration concept does not consider that the formation or encoding of a new memory relies on a time dependent process and implicitly supposes that the memory fixation occurs simultaneously with the training episode (Miller et al., 1986). Once again, this particular point needs further investigation but there are findings in the literature, obtained both in human and animals, which indicate that integration of new information can be seen very rapidly (Gordon and Feldman, 1978; Stark et al., 2010). According to what can be expected from brain performance, the integration concept suggests that memories are rapidly formed.

### State at the Time of Testing Determines Retrieval

The consolidation/reconsolidation hypothesis focuses almost entirely on the encoding phase and on the underlying process. The quality of the retention performance is mainly considered to be determined by the degree of success of this first stage. The consolidation hypothesis can take into account events delivered before training, such as those responsible for state dependency. However, events delivered after training which alter retention performance, are always considered to act on the consolidation processes.

According to the integration view, the quality of the retention performance depends on the matching between the training and the testing conditions. The matching not only refers to what occurs during training and testing, but also to experiences occurring around these critical periods. These periods are susceptible to the introduction of non-related information during encoding (such as amnesic agents), and thus affect the ability to retrieve the initial memory at the time of testing. Hence, the state prevailing at the time of testing largely determines the quality of the retention performance. From personal experience we all know that a specific memory might be inaccessible at a time and easily retrieved at another one. Hence the quality of the retention performance does not only rely on encoding processes but also largely on retrieval processes (Bartlett, 1932; Sara, 2000). It has been shown that memories of life experiences that are consistent in emotional quality with an individual’s mood state are more accessible to retrieval; that is, they have a higher probability of retrieval in free recall or can be retrieved more quickly (Bower, 1981; Greenberg et al., 2012). We have discussed in the above section how the internal state may affect the quality of the retention performance. The state at the time of retrieval is thus a determinant orienting our retrieval process towards memories that are more compatible with the present situation of the individual. Such a basic and implicit process could be very helpful to sort out memories and to target the most appropriate one to be reactivated in that particular circumstance. According to the integration view, the retention performance is largely determined by the congruency of the individual’s state at the time of testing with that at encoding.

### Memory Integration for New Therapeutic Approaches

The consolidation/reconsolidation hypothesis theoretically offers the possibility to erase remote memories. However, as previously mentioned, attempts to achieve that aim are worrisome and the findings remain inconclusive. The integration concept provides two different possibilities that may be useful for therapy: state dependency, and emotional remodeling.

#### State Dependency

Interestingly, the capacity of memory to integrate new information in a memory in order to render it less retrievable can possibly be used for therapeutic purpose. We have seen that modifying the internal state of a subject at the time of reactivation of a remote memory can induce difficulties to retrieve this memory when the subject returns to its original state. Substantial evidence indicates that this effect can induce strong performance disruption in rats. We can imagine that similar procedure might be very effective in disrupting the retrieval of pathological memories. To be effective, changes of state must be relatively important and thus the treatment should induce a highly salient internal milieu. For example, delivering treatments inducing nauseous feelings just before the reactivation of the pathological memory would be one strategy (Gisquet-Verrier et al., 2015). Indeed, the unintended amnesic effects reported after chemotherapy for cancer treatments in humans might be explained by this idea (Lindner et al., 2017). One may also propose administering drugs or treatments that very temporarily (during the reactivation of the pathological memory) modify the state of consciousness. This approach is currently being investigated for post traumatic stress disorder (PTSD) using MDMA (Feduccia and Mithoefer, 2018; Mithoefer et al., 2018). Furthermore we suggest that the altered state of consciousness may also account for the efficacy of treatments such as hypnosis, EMDR and perhaps for the dissociative amnesia often reported following extreme situations (Radulovic et al., 2018).

#### Emotional Remodeling

The malleability of memory might be used clinically to decrease the emotional value of pathological memories. This possibility was recently used to treat PTSD in an animal model by reactivating the trauma memory while the possibility of expressing an emotional response was reduced by a pharmacological treatment, a paradigm that we termed “emotional remodeling.” More specifically, we showed that delivering drugs such as amphetamine or oxytocin, before the reactivation of the trauma memory, may abolish PTSD symptoms in trauma vulnerable rats (Toledano and Gisquet-Verrier, 2014; Le Dorze et al., submitted). We proposed that, when administered prior to a reactivation of the trauma experience, these treatments strongly reduced the emotional component of that memory. Due to the malleability of the reactivated memory, this reduced emotional valence could be integrated within the trauma memory, decreasing its negative consequences. Emotional remodeling may also explain the effects of propranolol on PTSD or on drug-related memories. Researchers supporting the consolidation hypothesis proposed that propranolol induces a reconsolidation blockade (Dębiec and Ledoux, 2004; Soeter and Kindt, 2010; Brunet et al., 2018). However, since propranolol is also well known as an anxiolytic (Granville-Grossman and Turner, 1966; Tyrer and Lader, 1972), this drug can reduce the emotional response elicited by the reactivation of pathological memories, thereby decreasing its pathological aspect. We did obtain some encouraging results with an emotional remodeling under propranolol on a cocaine patient (Chopin et al., 2016). However, our prediction is that more definitive results could be obtained with a treatment that is more strongly targeted on the emotional component of the memory.

It must be mentioned that emotional remodeling is in fact not so new, as it is close to other therapeutic approaches, exploiting other processes, but based on relatively similar paradigms such as counterconditioning, exposure therapy, false memory, hypnosis, and neurolinguistic programming therapy (Bouton, 2002; Loftus and Davis, 2006; Foa, 2011; Sturt et al., 2012; Lee et al., 2017).

Hence the integration concept proposes new ways to treat pathological memories, which, may also help explain the efficacy of already used treatments. It also suggests the possibility of considering new treatments such as the use of the combinations of reactivation and drugs that may be more effective than those currently used.

## Consequences and Perspective

The integration concept is a new hypothesis, which seems to account well for numerous results. This view proposes a more dynamic conception of memory than the consolidation/reconsolidation hypothesis and appears to be more in agreement with what we could expect about brain functioning. However, this view indicates limits in our current knowledge and requires further experimental support.

In the next few years we will have to address a series of questions that remain open for investigation. In particular, we have to understand the processes, by which a memory goes from an active to inactive state (and the reverse), to determine other forms of potential support of memories, and to learn more about the nature of the integration process, among other things.

In conclusion, we hope that this article will stimulate research to test the validity of the integration concept and to answer some of the different points raised above.

## Author Contributions

Both authors listed have made substantial, direct and intellectual contribution to the work, and approved it for publication.

## Conflict of Interest Statement

The authors declare that the research was conducted in the absence of any commercial or financial relationships that could be construed as a potential conflict of interest.

## Footnotes

^1^Recent views on the consolidation hypothesis also consider that reactivated memories are malleable, but in a quite different way, as integration is restricted to new information allowing the updating of the remote memory.
